# Quantifying the clinical virulence of *Klebsiella pneumoniae* producing carbapenemase *Klebsiella pneumoniae* with a *Galleria mellonella* model and a pilot study to translate to patient outcomes

**DOI:** 10.1186/1471-2334-14-31

**Published:** 2014-01-15

**Authors:** Milena M McLaughlin, M Renee Advincula, Michael Malczynski, Grace Barajas, Chao Qi, Marc H Scheetz

**Affiliations:** 1 Department of Pharmacy Practice, Midwestern University Chicago College of Pharmacy, 555 31st Street, Downers Grove, IL 60515, USA; 2 Northwestern Memorial Hospital, 251 E Huron St, Chicago, IL 60611, USA; 3 Midwestern University, Chicago College of Pharmacy, 555 31st Street, Downers Grove, IL 60515, USA; 4 Jesse Brown VA Medical Center, 820 S Damen Ave, Chicago, IL 60612, USA; 5 Northwestern University, 710 N Lake Shore Dr, Chicago, IL 60611, USA

**Keywords:** *Klebsiella pneumoniae* producing carbapenemase *Klebsiella pneumoniae*, *Galleria mellonella*, Virulence, Mortality, Resistance

## Abstract

**Background:**

Previous studies may have overestimated morbidity and mortality due to *Klebsiella pneumoniae* producing carbapenemase (KPC) *Klebsiella pneumoniae* infections because of difficulties in modeling patient comorbidities. This pilot study sought to evaluate KPC virulence by combining clinical and *Galleria mellonella* models in patients with *K. pneumoniae* blood stream infections (BSIs).

**Methods:**

*G. mellonella* were inoculated using KPC(+) and KPC(−) isolates from these patients. Extent and rapidity of insect mortality was analyzed. Patients were stratified by KPC BSI status. Clinical outcomes of mortality and length of stay post-infection for survivors (LOS) were analyzed. Median virulence scores calculated from the insect studies were imputed in the clinical model.

**Results:**

The in-vivo model revealed greater mortality in KPC(−) isolates (p < 0.001). Fifteen patients with KPC(+) BSI were matched with 60 patients with KPC(−) BSI. Hospital mortality was greater in the KPC(+) group versus the KPC(−) group (OR 3.79, 95% CI 1.00 - 14.34). LOS was longer in the KPC(+) group (p < 0.01). Conversely the virulence score attenuated the association between KPC(+) status and mortality and LOS in the final translational models.

**Conclusions:**

KPC(+) status was associated with decreased virulence in GM. Opposite findings were observed in patients. This pilot study demonstrates that measured virulence from GM may differ from human estimates of virulence.

## Background

*Klebsiella pneumoniae* producing carbapenemase (KPC) *Klebsiella pneumoniae* are an emerging pathogen with the propensity to cause poor patient outcomes and high mortality [1]. There is controversy surrounding the reasons for these poor outcomes, including debate as to whether carriage of the resistance gene makes the organism more or less virulent. Clinical studies of KPC virulence to date have found discordant estimates of pathogenicity [2-6]. Furthermore, difficulties in modeling complex patient factors may have confounded the ability to discern effects on attributable mortality. The magnitude of patient mortality attributed to KPC may have also differed due to various study designs [7].

To attempt to obtain a more reliable estimate of the magnitude of KPC virulence, we created a pilot study that employed a validated *Galleria mellonella* host-pathogen interaction model using blood stream infection (BSI) isolates from KPC(+) and KPC(−) patients. *Galleria mellonella* is the caterpillar of the Greater Wax Moth and is well validated as an in-vivo model because of its humoral and cellular immune response pathways [8] and a clear correlation of organism virulence with mammalian models [9-11]. Furthermore, the organism *K. pneumoniae* has been extensively validated in the *G. mellonella* model in recent efforts [11,12]. Advantages of the *G. mellonella* model include the ability of *G. mellonella* to be injected with precise inocula and to be maintained at temperatures that mimic human host conditions [9,13-17]. Since the insects can be infected prospectively and randomly, this insect model also eludes the need to correct for disproportionate and complex patient comorbidities between KPC(+) and KPC(−) groups.

This novel study methodology study sought to quantitatively evaluate KPC virulence using a clinical translational approach: first, by assessing the relative virulence of representative KPC(+) and KPC(−) *K. pneumoniae* isolates from the clinical study with the *G. mellonella* host-pathogen interaction model; second, by determining the clinical virulence of KPC(+) *K. pneumoniae* blood stream infections by comparing KPC(+) to KPC(−) blood stream infections while controlling for confounding variables with standard clinical data modeling techniques; and third, by using the virulence score obtained from the insect model to re-inform the clinical model to obtain a corrected effect of KPC(+) virulence. It should be noted that the last effort is pilot in nature.

## Methods

### Organism identification and susceptibility testing

Organism identification was completed using the Vitek II system (bioMérieux, Balmes-les-Grottes, France) and manual biochemical identification when necessary. Antimicrobial susceptibility testing was performed on all isolates by the Vitek II system or Etest (AB bioMérieux, Solna, Sweden). Susceptibilities were categorized according to Clinical and Laboratory Standards Institute (CLSI) guidelines [18]. Isolates were classified as KPC(+) or KPC(−), with KPC(−) isolates being pan-susceptible except for ampicillin. KPC positive or negative status was confirmed using common primers for KPC 1, 2, and 3 (forward primer: 5′-TGT CAC TGT ATC GCC GTC-3′, reverse primer: 5′-CTC AGT GCT CTA CAG AAA ACC-3′).

### Molecular typing by pulsed field gel electrophoresis

Pulsed field gel electrophoresis (PFGE) was performed on all *K. pneumoniae* isolates to identify potential clonal spread. Genetic similarities of intra-patient isolates were determined by visual inspections of DNA banding patterns using the criteria of Tenover et al. [19] with isolates having 3 or fewer differing bands labeled as closely related, those with 4 to 6 differing bands considered possibly related, and those with more than 6 bands different considered genetically distinct.

### Galleria mellonella model

Each KPC(+) bloodstream isolate from the clinical study was randomly matched to one KPC(−) isolate from the clinical study. Twelve randomly selected insects weighing between 250–350 mg were selected for each isolate (Vanderhorst, Inc, St. Mary’s, OH). The insects were inoculated by injecting 1 x 10^6^ CFU per 10 microliter aliquot into the hemoceol via the rear left proleg using a 10 μL Hamilton animal syringe (Hamilton Co., Reno, NV, USA). Colony counts were performed by serial dilution with ultimate plating on blood agar (Remel, Lenexa, KS). Individual colonies were enumerated after 18–24 hours incubation at 38°C in ambient air. Any experiment with a colony count outside of a half log_10_ deviation was repeated. Phosphate buffer solution (placebo) injection controls and controls receiving no injection were used to evaluate trauma and attrition, respectively. Results were not included if greater than or equal to two insects died in either of the control groups [15]. The insects were incubated at 37°C in atmospheric air and observed every 24 h for 5 days. One common isolate was used for each experiment to control for inter-experiment insect robustness. Experiments were performed in duplicate and repeated in the case of discordance. Representative results are reported for all final experiments meeting inclusion criteria.

### Clinical study design and patient selection

A combined clinical and translational study comprised of a retrospective clinical study and an insect virulence model was conducted at Northwestern Memorial Hospital in Chicago, IL. Patients at least 18 years of age and with at least one positive blood culture for *Klebsiella pneumoniae* identified from March 2010 through December 2011 were eligible. Only the first isolate per patient during the study period was considered for inclusion. Four control KPC(−) patients were matched to each KPC(+) patient in order to optimize the relative gains in power associated with each additional patient included [20,21]. This study was reviewed and approved by the Northwestern Memorial Hospital and Midwestern University Institutional Review Boards with a waiver of informed consent.

### Patient data collection

Patient demographics and data variables were collected using inpatient electronic medical records, pharmacy databases, and clinical microbiological databases. Patient demographics included age, gender, modified Acute Physiology and Chronic Health Evaluation (APACHE) II score [22,23], prior hospitalizations, absolute neutrophil count (ANC) of less than 500 cells/mm^3^, renal dysfunction, hepatic dysfunction, diabetes, current immunosuppression, and length of stay prior to positive culture. Other clinical variables included time to directed therapy, number of clinical sites with positive cultures for *K. pneumoniae*, and total days of antibiotics.

### Clinical definitions

Hepatic dysfunction was defined as any liver function test value greater than 3 times the upper limit of normal at the time of culture [24]. Renal dysfunction was defined as chronic kidney disease, dialysis, or an increase in serum creatinine of 0.5 mg/dL or 50% at the time of culture as compared to a previous value [25,26]. Current immunosuppressive therapy was defined as the patient receiving therapy with any corticosteroids or chemotherapy at the time of admission. Since optimal and appropriate therapy is not fully elucidated for KPC, we assessed adequacy in several manners. Directed therapy was defined as any antibiotic given to a patient in a directed manner after culture results were available. Time to directed therapy was defined as the time in hours from the culture draw to the receipt of antibiotic therapy. As in-vitro activity of antibiotics is not entirely predictive of in-vivo outcomes for carbapenemase producing organisms, we assessed all drugs given in a directed manner (e.g. meropenem could be directed treatment for a KPC producing organism) [27,28].

### Statistical analysis

#### Galleria mellonella model analysis

The primary outcome for the insect model was rapidity and extent of mortality of the *G. mellonella* assessed with Kaplan-Meier survival curves and log rank tests.

### Clinical analysis

The outcomes for the clinical model were: hospital mortality of patients according to KPC status, duration of infection, carbapenem use post culture, and length of stay post-infection for survivors. For bivariate comparisons, the Student’s t-test, Wilcoxan Rank Sum, Chi-square, and Fisher’s Exact tests were used as appropriate. Multivariate models were created to assess the independent primary modifier of KPC status on mortality and length of stay post-infection for survivors. To find the most parsimonious and explanatory model, relevant confounders were controlled using a forward stepwise procedure by adding variables with a plausible relationship to the dependent outcomes and significant at the p < 0.2 level in bivariate analysis. Variables of interest, KPC status and virulence score, were forced as initial bivariate comparisons. Models were assessed for optimization and compared against the model with one additional variable. For linear models, normalized variable versus residual plots were examined, and variables unduly influenced by one or two outliers (i.e. slope essentially defined by one or two points) were removed from the model. The removal of these variables was done to conservatively attempt to see if KPC status retained a significant relationship with length of stay post-infection for survivors after correcting for the virulence factor and other highly significant variables. For logistic regressions, the final model was the most parsimonious model that was significantly different from the others (i.e. objective factor ≥ 3.84 for models differing by one exploratory variable for logistic regression and an increase in the adjusted-r^2^ value for linear regression).

### Combined model analyses

To conduct the pilot analyses imputing virulence scores, multivariate models from the clinical study were re-informed using virulence scores obtained from the insect model (i.e. area under the individual insect mortality curve scores were calculated from the Kaplan-Meier curves). Resultant virulence scores could range from 0–60 dead insect days. On each day a total of 12 insects were eligible to be dead or alive. Therefore the maximum score is 60 (12 insects x 5 days; 100% insect mortality on day 1), and the minimum score is zero (0 insects x 5 days; 0% insect mortality at day 5). Thus, higher scores indicate greater virulence. All tests were two-tailed and significance was set at p < 0.05. All statistical analyses were performed with Intercooled Stata, version 11.1 (Statacorp, College Station, TX).

## Results

A total of 17 KPC(+) blood stream infections were identified during the study period and considered for inclusion. Of these cultures, 2 were duplicates and were excluded from the analysis. Therefore 15 patients with KPC(+) blood stream infections were matched with 60 patients with KPC(−) blood stream infections and were included in the analysis. All isolates were genetically distinct per PFGE analysis (data not shown).

### Insect model results

**Figure 1:**
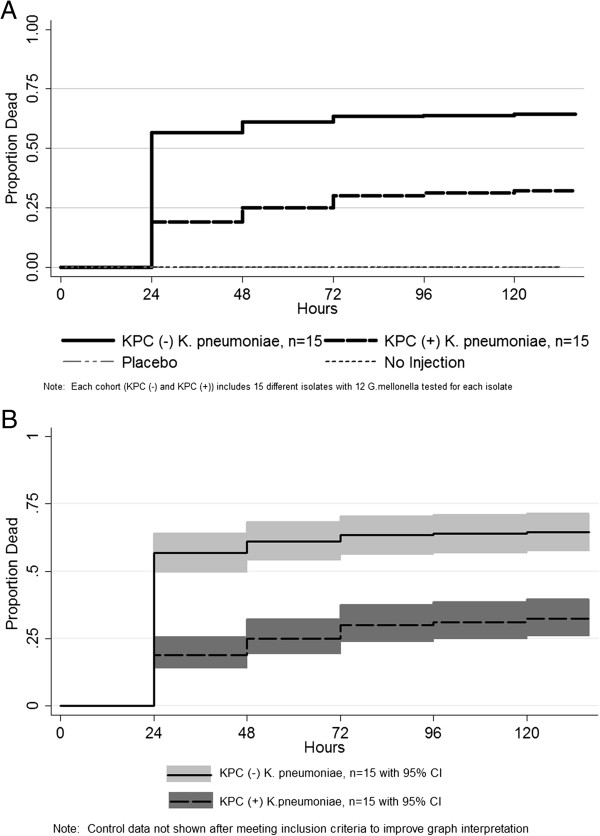
**Kaplan-Meier survival for *G. mellonella *inoculated with *K. pneumoniae *isolates showing mean values (1A) and 95% confidence intervals (1B).** KPC = *Klebsiella pneumoniae* producing carbapenemase.

In the *G. mellonella* model at 24 hours, KPC(−) *K. pneumoniae* isolates killed approximately 50% of the larvae compared with 25% mortality with larvae injected with KPC(+) *K. pneumoniae* isolates. This difference persisted throughout the five day study period (Figure 1). Accordingly differences in mortality over five days were observed between the KPC(+) and KPC(−) *K. pneumoniae* isolates (p < 0.001). The median virulence score for KPC(+) isolates was 13 insect days (IQR 3–23) and 50 insect days (IQR 31–56) for the KPC(−) isolates. A median virulence score of 50 insect days was utilized for the remaining 45 KPC(−) patients.

### Clinical results

The mean age, number of female patients, and modified APACHE II score were similar between the KPC positive and negative groups (Table 1). There were a larger percentage of patients receiving current immunosuppressive therapy in the KPC(−) group (18.3% vs. 6.7%), however, this was not statistically significant. A greater percentage of patients in the KPC(+) group had prior hospitalizations (80% vs. 46.7%, p = 0.02). The KPC(+) group also exhibited a significantly longer length of stay prior to positive culture, length of antibiotic treatment, and more frequent *K. pneumoniae* cultured from more than one site (p < 0.01 for all). No difference was found between the groups for time to directed therapy. Clinical characteristics and outcomes of KPC(+) patients are shown in Table 2.

**Table 1:** **Demographic and clinical characteristics of patients with *K. pneumoniae *** **blood stream infections stratified by KPC status**

**Characteristic**	**KPC(+) (n = 15)**	**KPC(−) (n = 60)**	**p-value**
Age (mean, SD)	59.5 (11.3)	59.6 (17)	0.98
Gender, female	32 (53.3)	8 (53.3)	0.99
Modified APACHE II score (mean, SD)	12.1 (5.4)	10.8 (4.2)	0.31
Prior hospitalization	12 (80)	28 (46.7)	0.02
Neutropenic	1 (6.7)	1 (1.7)	0.36
Renal dysfunction	3 (20)	10 (16.7)	0.72
Liver dysfunction	2 (13.3)	5 (8.3)	0.62
Diabetes	3 (20)	10 (16.7)	0.72
Current immunosuppressive therapy	1 (6.7)	11 (18.3)	0.44
LOS prior to positive culture (median, IQR)	2 (1–12)	1 (1–1)	<0.01
Time to directed therapy (hrs) (median, IQR)	4 (0–24)	6 (3–13)	0.46
*K. pneumoniae* cultured from >1 site	11 (73.3)	19 (31.7)	<0.01
Total days of antibiotics post infection (median, IQR)	13 (8–18)	6.5 (4–10)	<0.01

Note: Data are no. (%) of patients unless otherwise indicated.

KPC = *Klebsiella pneumoniae* producing carbapenemase, APACHE = Acute Physiology and Chronic Health Evaluation, LOS = length of stay, SD = standard deviation, IQR = interquartile range.

**Table 2:** Clinical characteristics and outcomes of KPC(+) patients

	**Patient characteristics**			**KPC susceptibilities (mg/L)**			**Patient treatment and outcome**	
**Age**	**Modified APACHE II score, day 0**	**Antibiotics received prior to culture**	**Bacteremia source**	**Ertapenem MIC**	**Imipenem MIC**	**Meropenem MIC**	Directed therapy*	**Outcome**
55	10	Vancomycin, tobramycin	Lung	>8	8	≥16	None	Died
79	18	Ciprofloxacin	PICC line, wound	>8	8	≥16	Meropenem	Died
68	13	Vancomycin, meropenem	Lung/GU	Not done	2	4	Meropenem	Died
65	22	Meropenem, cefepime, gentamicin, vancomycin	GU	>32	>32	>16	Cefepime, tigecycline, amikacin	Died
54	6	Piperacillin/tazobactam, vancomycin, tobramycin, meropenem, colistin	GU	Not done	8	≥16	Tobramycin, meropenem, colistin, tigecycline, gentamicin	Died
40	13	None	GU	≥8	≥16	≥16	Cefepime	Discharge home
38	11	Piperacillin/tazobactam, vancomycin, gentamicin, linezolid	Kidney	4	1	1	Cefepime, gentamicin, colistin	Discharge home
75	21	None	GI/GU	>32	4	2	Piperacillin/tazobactam, tigecycline, gentamicin, fosfomycin	Discharge home
64	9	Ciprofloxacin, meropenem, vancomycin, aztreonam, tigecycline	GU	32	16	2	Cefepime, fosfomycin, tigecylcine, colistin	Discharge home
59	10	None	GU	>8	4	2	Meropenem, colistin, amikacin, tigecycline, tobramcyin	Discharge home
58	13	Vancomycin, piperacillin/tazobactam, tigecycline	PICC line	32	16	Not done	Cefepime, amikacin	Transfer to LTAC
55	8	Vancomycin, meropenem	PICC line, wound	>8	>16	>16	Colistin, tigecycline, cefepime	Transfer to LTAC
64	10	None	Intraabdominal	12	4	1	Meropenem, tigecycline, imipenem/cilastatin, colistin, piperacillin/tazobactam	Transfer to LTAC
67	15	Cefepime, ciprofloxacin, gentamicin, linezolid	GU likely	>8	4	2	Cefepime, gentamicin	Transfer to rehab
52	2	Ciprofloxacin, cefazolin, vancomycin	Unknown	Not done	8	Not done	Piperacillin/tazobactam, ciprofloxacin, cefepime, gentamicin, colistin, meropenem, tigecycline	Transfer to rehab

GU = genitourinary, GI = gastrointestinal, PICC = peripherally inserted central catheter, LTAC = long term acute care, Rehab = rehabilitation organization.

*Directed therapy was defined as antibiotics received after culture results were available.

Previously hospitalized patients and those with renal dysfunction were less likely to survive their hospital stay (p < 0.01 for both; Table 3). The hospital length of stay prior to positive culture was also longer in the non-survivor group (p < 0.01). The median time to directed therapy was zero days (interquartile range [IQR] 0–5.25) in the non-survivors and 6 days (IQR 3–15.5) in the survivor group (p < 0.01).

**Table 3:** **Demographic and clinical characteristics of patients with *K. pneumoniae *** **blood stream infections stratified by survivors versus non-survivors**

**Variable**	**Survived (n = 63)**	**Died (n = 12)**	**p-value**
Age (mean, SD)	58.8 (16.6)	64 (11.7)	0.30
Gender, female	32 (50.8)	8 (66.7)	0.36
Modified APACHE II score (mean, SD)	10.6 (4.3)	13.2 (4.9)	0.07
Prior hospitalization	29 (46)	11 (91.7)	<0.01
Neutropenic	2 (3.2)	0 (0)	0.99
Renal dysfunction	7 (11.1)	6 (50)	<0.01
Liver dysfunction	6 (9.5)	1 (8.3)	0.99
Diabetes	7 (11.1)	4 (33.3)	0.07
Current immunosuppressive therapy	10 (15.9)	2 (16.7)	0.99
LOS prior to positive culture (median, IQR)	1 (1–1)	10.5 (2.5-23)	<0.01
Time to directed therapy (hrs) (median, IQR)	6 (3–15.5)	0 (0–5.25)	<0.01
*K. pneumoniae* cultured from >1 site	24 (38.1)	6 (50)	0.44
Total days of antibiotics post infection (median, IQR)	7 (4–14)	9 (5–15.5)	0.56
KPC(+) status	10 (15.9)	5 (41.7)	0.055

Note: Data are no. (%) of patients unless otherwise indicated.

KPC = *Klebsiella pneumoniae* producing carbapenemase, APACHE = Acute Physiology and Chronic Health Evaluation, LOS = length of stay, SD = standard deviation, IQR = interquartile range.

The outcome of hospital mortality was greater in the KPC(+) group (n = 5, 33.3%) versus the KPC(−) group (n = 7, 11.7%; p = 0.055; Table 4); however, this difference narrowly failed to meet the threshold for statistical significance. There was also a larger percentage of KPC(+) patients that received a carbapenem post culture (n = 6, 40%) than the KPC(−) group (n = 4, 6.7%; p < 0.01). The median length of stay post infection for survivors was 18.5 days (IQR 15–22) in the KPC(+) group compared with 7 days (IQR 6–13) in the KPC(−) group (p < 0.01). The duration of infection did not differ significantly between the groups.

**Table 4:** **Outcomes of patients with *K. pneumoniae *** **blood stream infections stratified by KPC status**

**Variable**	**KPC(+) (n = 15)**	**KPC(−) (n = 60)**	**p-value**
In-hospital mortality	5 (33.3)	7 (11.7)	0.055
Duration of infection (median, IQR)	3 (3–4)	3 (3–5)	0.74
Carbapenem used post culture	6 (40)	4 (6.7)	<0.01
LOS post infection, survivors (median, IQR)	18.5 (15–22)	7 (6–13)	<0.01

Note: Data are no. (%) of patients unless otherwise indicated.

KPC = *Klebsiella pneumoniae* producing carbapenemase, IQR = interquartile range.

### Multivariate and translational pilot model results

**Figure 2:**
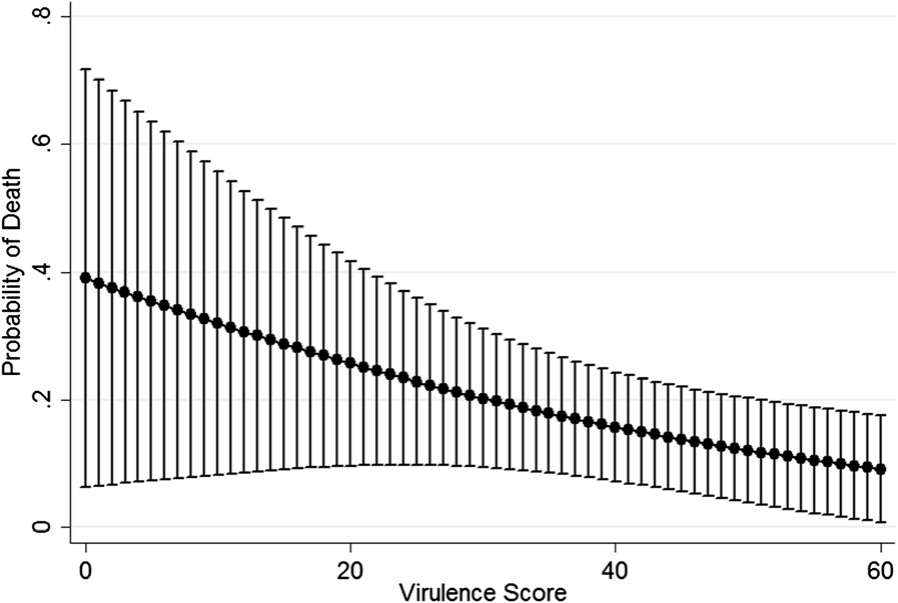
**Probability of patient death according to *Galleria mellonella *** **virulence score.**

For the outcome of mortality, in the first multivariate model, KPC(+) status was associated with increased odds of mortality, (OR 3.79, 95% CI 1.00 – 14.34; Table 5). In the second multivariate model, modified APACHE II score was associated with a 13% nonsignificant increase in mortality for each increase of 1 in APACHE II score (OR 1.13, 95% CI 0.97-1.32), and KPC(+) status had a similar, but less significant association (OR 3.34, 95% CI 0.84-13.27) relative to the first model. In the third model, virulence score attenuated the association between KPC(+) status and mortality (OR 2.51, 95% CI 0.2-31.1). When virulence score was assessed alone with mortality, each increase of virulence score was associated with a downward trending 3% drop in mortality (0.97, 95% CI 0.94-1.00). Probabilities of death calculated from the odds ratio can be found in Figure 2.

**Table 5:** **Multivariate analyses for risk factors for in-hospital mortality of patients with *K. pneumoniae *** **blood stream infections**

**Model**	**Variable**	**Mortality OR (95% Confidence interval)**	**p-value**
1	KPC(+) status	3.79 (1.00 - 14.34)	0.05
2	KPC(+) status	3.34 (0.84 - 13.27)	0.09
	Modified APACHE II	1.13 (0.97 - 1.32)	0.12
3	KPC(+) status	2.51 (0.20 - 31.14)	0.48
	Modified APACHE II	1.13 (0.97 - 1.32)	0.13
	Virulence score	0.99 (0.93-1.06)	0.79

KPC = *Klebsiella pneumoniae* producing carbapenemase, APACHE = Acute Physiology and Chronic Health Evaluation.

**Figure 3:**
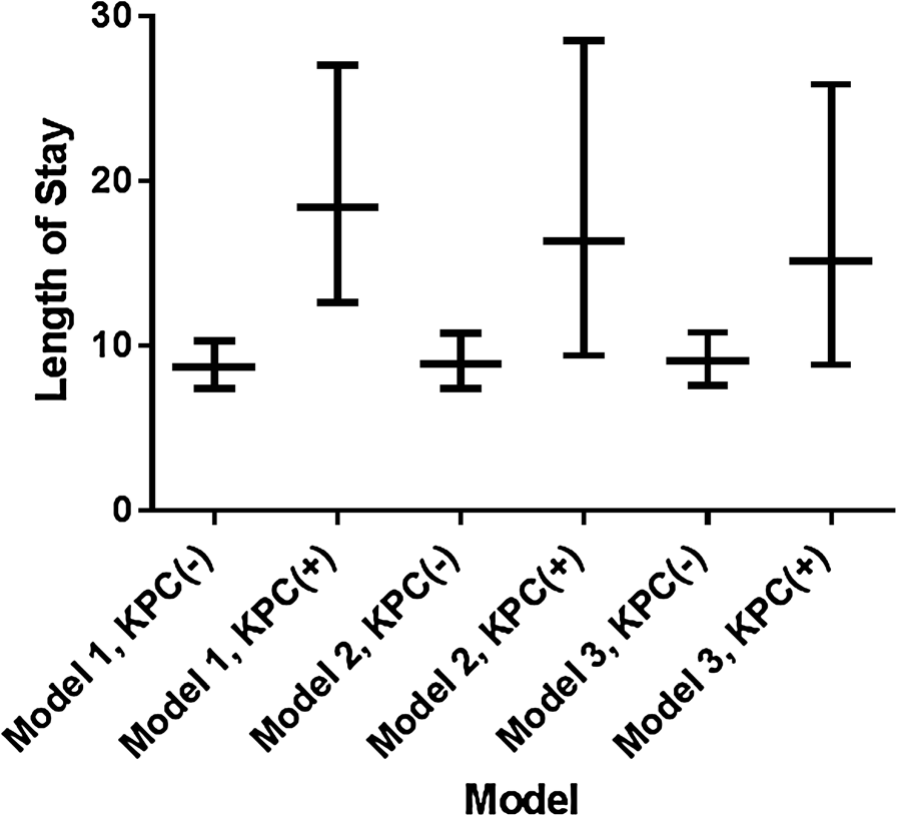
**Multivariate analysis adjusted length of stay post infection (mean, 95% CI) for KPC(+) and KPC(−) *K. pneumoniae *blood stream infections.** *Length of stay post infection for survivors was modeled as a natural log transformed variable. It is reported as inverse natural log for ease of interpretation. **Model 1: Bivariate LOS for KPC(+) and KPC(−) patients; Model 2: LOS for KPC(+) and KPC(−) patients, adjusted for insect virulence score; Model 3: LOS for KPC(+) and KPC(−) patients adjusted for insect virulence score, and transfer to ICU post culture. KPC = *Klebsiella pneumoniae* producing carbapenemase, LOS = length of stay.

For the outcome of length of stay post-infection for survivors, in the first multivariate model, KPC(+) status was associated with an increased length of stay (2.11 days, 95% CI 1.38-3.23, p < 0.01). This association did not remain significant when the virulence score was added to the model (1.83 days, 95% CI 0.96-3.5, p = 0.066) or when variables were added to the model according to the prespecified building criteria (1.67 days, 95% CI 0.89-3.12, p = 0.11). As additional relevant variables were added to the model, the significance between the mean lengths of stay between KPC(+) and KPC(−) groups diminished as demonstrated in Figure 3.

## Discussion

This study found decreased virulence for KPC (+) isolates compared to KPC(−) isolates in a *G. mellonella* model. The study analyzing patient outcomes from the patients that contributed the isolates found the opposite association at the bivariate level (i.e. KPC (+) patients experienced greater mortality). Specifically, this clinical study identified a 20% absolute increase in hospital mortality for KPC(+) patients. Minimally, the discordances between these two models should give researchers pause. Our pilot study imputing a virulence score, which should be interpreted with caution due to low power, demonstrated that attributable virulence in patients was decreased when considering the insect model virulence results. In a separate analysis designed to limit the number of predictor variables entered into the multivariate logistic model, increasing organism virulence score from the *G. mellonella* model was associated with downward trending mortality rates at the bivariate level. The length of stay post-infection for survivors was 11.5 days longer in the KPC(+) group when considering the unadjusted model. Similarly here, the magnitude of difference and association was attenuated with the addition of the virulence score. The *G. mellonella* model indicated that KPC(−) *K. pneumoniae* isolates were more virulent than KPC(+) *K. pneumoniae* isolates.

*G. mellonella* larvae have been shown as a validated invertebrate host model for deciphering virulence in *K. pneumoniae*[11,12] as well as a number of other bacterial pathogens including: *Listeria* spp. [29], *Staphylococcus aureus*[16], *Acinetobacter baumannii*[15], *Pseudomonas aeruginosa*[30], among others. Several studies have also used the *G. mellonella* model to demonstrate the virulence between different strains or genetic mutations of organisms [11,15,16]. Results from this host-pathogen interaction model have also been shown to produce similar results to mouse models with *K. pneumoniae*[11]. Importantly known *K. pneumoniae* virulence factors such as capsule polysaccharide and biochemical manipulations to Lipid A when studied as knock-out mutants and compared to isogenic parent isolates, demonstrate that *G. mellonella* models are highly capable of discerning virulence [11]. Concordance has been seen with other organisms using virulence models with *G. mellonella* and higher order animal studies. Wand et al. studied the virulence of several species of *Burkholderia* and found that the results found in the *G. mellonella* model reflected those found in a similar murine infection model [31]. Jander et al. reported a positive correlation (p < 0.001) of virulence patterns between *Pseudomonas aeruginosa* isolates tested in *G. mellonella* and a burned mouse model [10].

Prior work, as described below, suggests that patients with KPC(+) *K. pneumoniae* blood stream infections have a higher mortality than patients with KPC(−) isolates. Based upon the results of this study, the higher mortality may be due to patient factors and not virulence of the resistant organism. In the *G. mellonella* model, KPC(+) *K. pneumoniae* isolates were less virulent than KPC(−) isolates; there may be a fitness cost to carriage of the resistant plasmid, or less fit organisms might be more likely to acquire the resistance. While additional study is certainly needed, the results of this pilot study suggest that it might not be the virulence of KPC(+) *K. pneumoniae* that leads to poor patient outcomes.

Previous studies have evaluated outcomes of patients with KPC(+) *K. pneumoniae* blood stream infections; however, these studies used study designs different from ours. Borer et al. conducted a matched, retrospective, cohort study and found a 50% attributable mortality rate for KPC(+) *K. pneumoniae* blood stream infections when compared to hospitalized patients that were infected but without bacteremia [3]. In a case–control study evaluating patient outcomes, Mouloudi et al. reported in-hospital mortality of 68% in the KPC(+) *K. pneumoniae* blood stream infection group compared to 41% in the carbapenem susceptible *K. pneumoniae* group and 44% in the metallo-beta-lactamase producing *K. pneumoniae* blood stream infection group [4]. Similarly, Ben-David et al. conducted a retrospective cohort study of patients with KPC(+) *K. pneumoniae* blood stream infections compared to extended spectrum beta-lactamase (ESBL) producing *K. pneumoniae* blood stream infections and carbapenem susceptible *K. pneumoniae* blood stream infections [2]. The authors found that infection-related mortality was significantly higher among patients with KPC(+) infections compared to ESBL producing or carbapenem susceptible infections (48%, 22%, and 17%, respectively). All three of these studies have found a higher mortality rate in carbapenem resistant blood stream infections, but like all clinical outcomes studies, they may suffer from an inability to completely correct for all comorbidity variables. Our use of the *G. mellonella* model allowed for KPC status to be isolated as the only changing variable.

This study analyzed the virulence of *K. pneumoniae* based on KPC status as a clinical variable and has shown that KPC(+) status was not associated with mortality when the virulence score was included in the model. Virulence was directly calculated from a translational in-vivo model in which all variables were held constant except for KPC status. Prospective group assignment in the insect model allowed unknown confounders to be balanced between the groups and was able to provide an unbiased estimate of the effect of KPC status.

Studies conducted by Zarkotou et al. [6] and Tumbarello et al. [5] both found that increasing APACHE scores were risk factors for mortality in patients with KPC(+) *K. pneumoniae* blood stream infections. This would indicate that patient factors other than resistance may drive poor patient outcomes. Similarly, Ben-David et al. and Mouloudi et al. reported that increasing Pitt bacteremia score, Charlson score, and Sequential Organ Failure Assessment (SOFA) score were independent risk factors for mortality, respectively [2,4]. Both of these studies also found that carbapenem resistance was an independent risk factor for mortality. These studies suggest that KPC(+) *K. pneumoniae* are being isolated from critically ill patients, and it may be impossible with conventional clinical study methodologies to statistically separate out the attributable virulence from KPC status.

One of our statistical findings may require additional explanation. The bivariate logistic regression model, utilizing the Chi-square test, identified a significant effect of KPC on mortality (p = 0.050). The same data analyzed with the more conservative Fisher’s Exact test resulted in findings with p = 0.055. While bivariate logistic regression is appropriate for multivariate model building, the Fisher’s Exact test is mathematically most appropriate. Despite interpretive differences, the findings demonstrate a trend towards statistical significance that is consistent with previous literature reporting that KPC(+) status portends increased mortality. Had our sample size been larger, the increased study power would have likely resulted in statistical significance (i.e. p < 0.05) regardless of the test used.

While we believe that this novel methodology can improve effect estimates in statistically complex clinical data models, this pilot study has limitations that must be considered. First, the clinical study was retrospective and had a limited number of patients available. Still, the *G. mellonella* studies demonstrated decreased virulence for KPC(+) strains), and minimally the results demonstrate discordance between the models. Our imputation model attempted to combine these results, but care should be taken in interpreting these results until a larger study can confirm results. The limited patient enrollment concern was partially addressed by matching KPC(+) patients on a 1:4 basis with KPC(−) patients in order to increase the sample size, yet numbers were constrained. Unfortunately, the model cannot easily be improved by simply adding patients. Our data are highly unique because KPC blood infections remain a rare event. For instance, the CDC estimated that there are only 9,300 cases [32] of carbapenem-resistant Enterobacteriaceae infections in the United States per year. Only a fraction of these are KPC and even a smaller fraction is bloodstream isolates. Thus our numbers are quite large for a single center study. To further illustrate this point, 386 Enterobacteriaceae blood stream infections occurred at our 897-bed, tertiary-care, academic center in 2012. Of these, only 6 isolates were KPC. Multicenter trials may be necessary for future analyses.

Second, there may have been inter-experimental variation in the robustness of the *G. mellonella*; however, we accounted for this by using an inter-experimental control isolate to in order to adjust for any differences in insects. Third, the potential exists for differences in regional strains of *K. pneumoniae*; a larger multi-center study could address organism epidemiology. Fourth, it is possible that the *G. mellonella* results may not correlate with human outcomes, but this possibility is less likely given the translatability of the *G. mellonella* model with other bacterial virulence studies [12-17] and animal studies [10,11]. Fifth, optimal therapy for these infections has not been fully defined, and the effect of antibiotic therapy on survival is difficult to assess in this study due to limited power. Finally, due to 1:1 matching of isolates for the insect model, median virulence scores were extrapolated for 45 patients. However, the findings obtained when we analyzed a subset of 1:1 matched patients (i.e. patients for which actual virulence scores were obtained and not calculated) were concordant to those of the entire cohort (data not shown).

## Conclusions

In this study, results from the insect model indicated that KPC(+) *K. pneumoniae* isolates were less virulent than KPC(−) *K. pneumoniae* isolates. KPC(+) status was associated with a 20% absolute increase in hospital mortality for patients at the bivariate level. This finding was attenuated when measured insect virulence was imputed into the model. Similarly, the length of stay post- infection for survivors was significantly longer in the KPC(+) group, but again the virulence score attenuated the association. Our novel pilot methodology suggests that previous clinical studies may have overestimated virulence. There is a need for further studies with increased power to fully elucidate the clinical virulence of KPC(+) *K. pneumoniae*.

## Abbreviations

ANC: Absolute neutrophil count; APACHE: Acute physiology and chronic health evaluation; BSI: Blood stream infection; CI: Confidence interval; ESBL: Extended spectrum beta-lactamase; KPC: *Klebsiella pneumoniae* producing carbapenemase; LOS: Length of stay; OR: Odds ratio; PFGE: Pulsed field gel electrophoresis.

## Competing interests

Authors of this manuscript have the following to disclose concerning possible financial or personal relationships with commercial entities that may have a direct or indirect interests in the subject matter of this presentation: All authors: no relevant conflicts.

## Authors’ contributions

MMM and MHS designed the study and performed the statistical analyses. MMM, MRA, and MHS acquired clinical data. MMM, MM, GB, QC, and MHS acquired laboratory data. MMM, MRA, and MHS drafted and critically revised the manuscript. All authors read and approved the final manuscript.

## Pre-publication history

The pre-publication history for this paper can be accessed here:

http://www.biomedcentral.com/1471-2334/14/31/prepub
